# GDF11 Modulates Ca^2+^-Dependent Smad2/3 Signaling to Prevent Cardiomyocyte Hypertrophy

**DOI:** 10.3390/ijms19051508

**Published:** 2018-05-18

**Authors:** Javier Duran, Mayarling Francisca Troncoso, Daniel Lagos, Sebastian Ramos, Gabriel Marin, Manuel Estrada

**Affiliations:** Programa de Fisiología y Biofísica, Instituto de Ciencias Biomédicas, Facultad de Medicina, Universidad de Chile, Santiago 8389100, Chile; javiadg@ug.uchile.cl (J.D.); mayarling.troncoso@gmail.com (M.F.T.); daniel.9um@gmail.com (D.L.); sebramos@udec.cl (S.R.); gf.marint@gmail.com (G.M.)

**Keywords:** growth differentiation factor 11, Ca^2+^ signaling, cardiomyocyte hypertrophy, Smad2/3 proteins

## Abstract

Growth differentiation factor 11 (GDF11), a member of the transforming growth factor-β family, has been shown to act as a negative regulator in cardiac hypertrophy. Ca^2+^ signaling modulates cardiomyocyte growth; however, the role of Ca^2+^-dependent mechanisms in mediating the effects of GDF11 remains elusive. Here, we found that GDF11 induced intracellular Ca^2+^ increases in neonatal rat cardiomyocytes and that this response was blocked by chelating the intracellular Ca^2+^ with BAPTA-AM or by pretreatment with inhibitors of the inositol 1,4,5-trisphosphate (IP_3_) pathway. Moreover, GDF11 increased the phosphorylation levels and luciferase activity of Smad2/3 in a concentration-dependent manner, and the inhibition of IP_3_-dependent Ca^2+^ release abolished GDF11-induced Smad2/3 activity. To assess whether GDF11 exerted antihypertrophic effects by modulating Ca^2+^ signaling, cardiomyocytes were exposed to hypertrophic agents (100 nM testosterone or 50 μM phenylephrine) for 24 h. Both treatments increased cardiomyocyte size and [^3^H]-leucine incorporation, and these responses were significantly blunted by pretreatment with GDF11 over 24 h. Moreover, downregulation of Smad2 and Smad3 with siRNA was accompanied by inhibition of the antihypertrophic effects of GDF11. These results suggest that GDF11 modulates Ca^2+^ signaling and the Smad2/3 pathway to prevent cardiomyocyte hypertrophy.

## 1. Introduction

Growth differentiation factor 11 (GDF11) is a member of the transforming growth factor-β (TGF-β) superfamily and has various roles in the cardiovascular system [1,2]. Circulating GDF11 levels increase after induction of cardiac infarction in mice [3]. Moreover, the degree of plasma GDF11 concentration increase is dependent on the severity of heart failure, suggesting that GDF11 is an adjustable mediator in cardiac remodeling [3]. Consequently, exogenous administration of GDF11, through controlled peptide delivery from secretory cells, decreases infarct size in the heart, which contributes to preservation of cardiac function [4]. Circulating GDF11 levels represent an important negative regulator of hypertrophic and remodeling processes, which contribute to heart failure. The highest levels of GDF11 have been related to a lower risk of cardiovascular events and death in humans [5]. In addition, exogenous administration of GDF11 blocks cardiac hypertrophy induced by various stimuli or prohypertrophic conditions [6]. Conversely, other reports have indicated that GDF11 does not prevent cardiac hypertrophy in a pressure overload model [6], and that GDF11 circulating levels increase with age and induce cardiac hypertrophy [7], as well as inhibiting skeletal muscle regeneration [8]. Although diverse effects of GDF11 have been reported in cardiac cells, the mechanisms underlying these effects are poorly understood.

Ca^2+^ signals are important effectors that facilitate normal cardiac function. In cardiomyocytes, Ca^2+^ plays major roles in excitation-contraction coupling and as a second messenger for numerous agonist-mediated Ca^2+^-dependent pathways. Moreover, Ca^2+^ has been shown to have critical roles in diverse cellular events, including differentiation, metabolism, apoptosis, and hypertrophy [9,10]. Cardiomyocyte growth is finely controlled by Ca^2+^-mediated signaling; however, it is still unclear whether the actions of GDF11 involve Ca^2+^-mediated pathways to induce negative regulation during cardiac hypertrophy. Recent studies have demonstrated that members of the TGF-β family induce Ca^2+^ increases by promoting Ca^2+^ release from intracellular stores [11,12]. GDF15, an antihypertrophic factor, increases *Ica* in neural cells, suggesting a relationship between proteins similar to GDF11 and Ca^2+^-dependent mechanisms [11]. Cardiac hypertrophy is a critical adaptive response of cardiomyocytes to preserve work output and efficiency of the heart [13]. Hypertrophic stimuli converge in common signaling pathways to induce cardiomyocyte growth, a process characterized by increased cell size and protein synthesis [14].

Although some studies have shown that GDF11 activates intracellular signaling pathways, such as those induced by myostatin [15], the intracellular pathways involved in antihypertrophic effects remain elusive. TGF-β-like proteins, such as GDF11, recruit and induce the phosphorylation of receptor-regulated Smad (from *Drosophila* homolog “mothers against decapentaplegic”, and the smooth muscle actin protein in *Caenorhabditis elegans*). Smad2 and Smad3 (Smad2/3) transduce extracellular signals from activin/TGF-β receptor ligands [16,17]. Smad2/3 proteins possess two globular domains associated by a linker region. The N-terminal domain (NH1 domain) has a region of nuclear localization and DNA binding. The C-terminal domain is highly conserved in all Smad proteins and can interact with numerous transcriptional factors [18]. The binding of a ligand to the type II receptor of TGF-β (activin receptor kinase IIA or IIB) recruits and phosphorylates TGF-β receptor dimer type I (activin-like receptor kinase 4/5 or 7) and forms a heterotetrametric complex to activate and phosphorylate Smad2 at residues Ser465/467 and Smad3 at residues Ser423/425 by the type I receptor [18]. Phosphorylation generates a secondary affinity site for the formation of a complex with Smad4; this complex then translocates to the nucleus and binds to specific protein cofactors, such as FoxH1 and p300 acetyltransferase, and to promoter regions in the DNA with a consensus sequence of 5′-GTCT-3′ through its C-terminal domain to control gene transcription [19].

GDF11 increases Smad2/3 phosphorylation in human embryonic stem cell-derived cardiomyocytes [6] and cardiac tissue [20]. Moreover, GDF11 induces nuclear translocation of Smad2/3 proteins in neuronal cells [21]. GDF8 or myostatin, a protein that shares 90% sequence identity in the mature state with GDF11, activates Smad2/3 in skeletal muscle cells, and both proteins are necessary to induce muscle atrophy [22]. Moreover, mice with knockout of Smad4—a mediator of Smad2/3 nuclear translocation—develop cardiac hypertrophy, whereas overexpression of Smad2 attenuates cardiomyocyte hypertrophy. Moreover, overexpression of the inhibitory Smad proteins Smad6/7 reverses the antihypertrophic effects of GDF15 [23]. Through the TGF-β type II receptor, TGF-β increases intracellular Ca^2+^ by activating L-type Ca^2+^ channels in rat neurons [11]. In pancreatic cancer cells, TGF-β induces increase of intracellular Ca^2+^ levels, an effect mediated by receptors for inositol 1,4,5-trisphosphate (IP_3_), which suppresses the activity of phosphatidylinositol-3,4,5-triphosphate 3-phosphatase and protein kinase C type α [24]. In addition, decreased Ca^2+^ levels inhibit the phosphorylation and nuclear translocation of Smad2/3 induced by TGF-β in fibroblasts [25]. This suggests that Ca^2+^ is involved in the activation of Smad2/3 in mammalian cells.

In this study, we examined the antihypertrophic effects of GDF11 through Smad2/3 proteins. Our results showed that the effects of GDF11 on cardiomyocytes involved subcellular trafficking and transcriptional activity of Smad2/3 proteins via Ca^2+^-dependent signaling.

## 2. Results

### 2.1. GDF11 Increased Intracellular Ca^2+^ in Neonatal Cultured Cardiomyocytes

**Figure 1:**
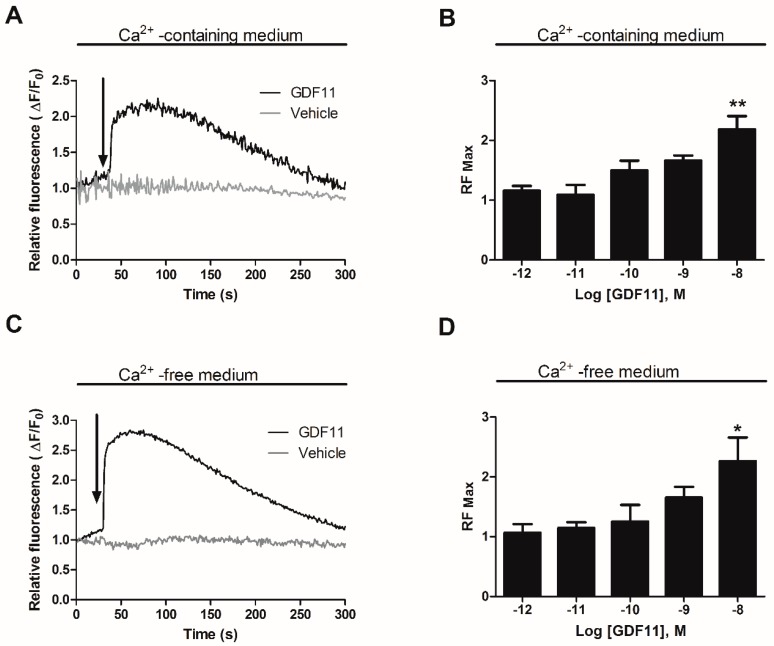
Effects of growth differentiation factor 11 (GDF11) on intracellular Ca^2+^ levels in cultured cardiomyocytes. Cells were preloaded with Fluo3-AM. The graphs show Ca^2+^-containing medium at the moment of stimulation. Intracellular Ca^2+^ levels were expressed as relative total fluorescence ratio (ΔF/F_0_) (**A**) Representative ΔF/F_0_ calculated from fluorescence images of cardiomyocytes treated with GDF11 (10 nM, black line); (**B**) Maximal relative fluorescence (RFmax) from all experiments performed with different concentrations of GDF11; or in Ca^2+^-free medium at the moment of stimulation; (**C**) Representative ΔF/F_0_ calculated from fluorescence images of cardiomyocytes treated with GDF11 (10 nM, black line); (**D**) RF max from all experiments performed with different concentrations of GDF1. Values are expressed as mean ± standard error of the mean (SEM) of triplicates from three independent experiments. Arrows indicate the time of addition of GDF11. * *p* < 0.05, ** *p* < 0.01 versus the control.

Fluctuations in intracellular Ca^2+^ levels were examined in cultured cardiomyocytes preloaded with Fluo3-AM. After addition of GDF11 (10 nM), the cardiomyocytes responded with an increase in free intracellular calcium by approximately 50% compared to initial levels (Figure 1A). The rise in Ca^2+^ levels was fast (5–10 s) and transient, returning to basal levels after 5 min of stimulation. The dose-response curve showed a peak at 10 nM GDF11 (Figure 1B). GDF11 did not induce detectable contraction of the cardiomyocytes. In Ca^2+^-free medium, a similar response pattern was observed with GDF11 (10 nM); this Ca^2+^ peak was transient and was reduced to baseline levels by 5 min post-stimulation (Figure 1C). Similar to the dose-response determined in Ca^2+^-containing medium, the dose-response curve in Ca^2+^-free medium showed a peak at 10 nM GDF11 (Figure 1D).

**Figure 2:**
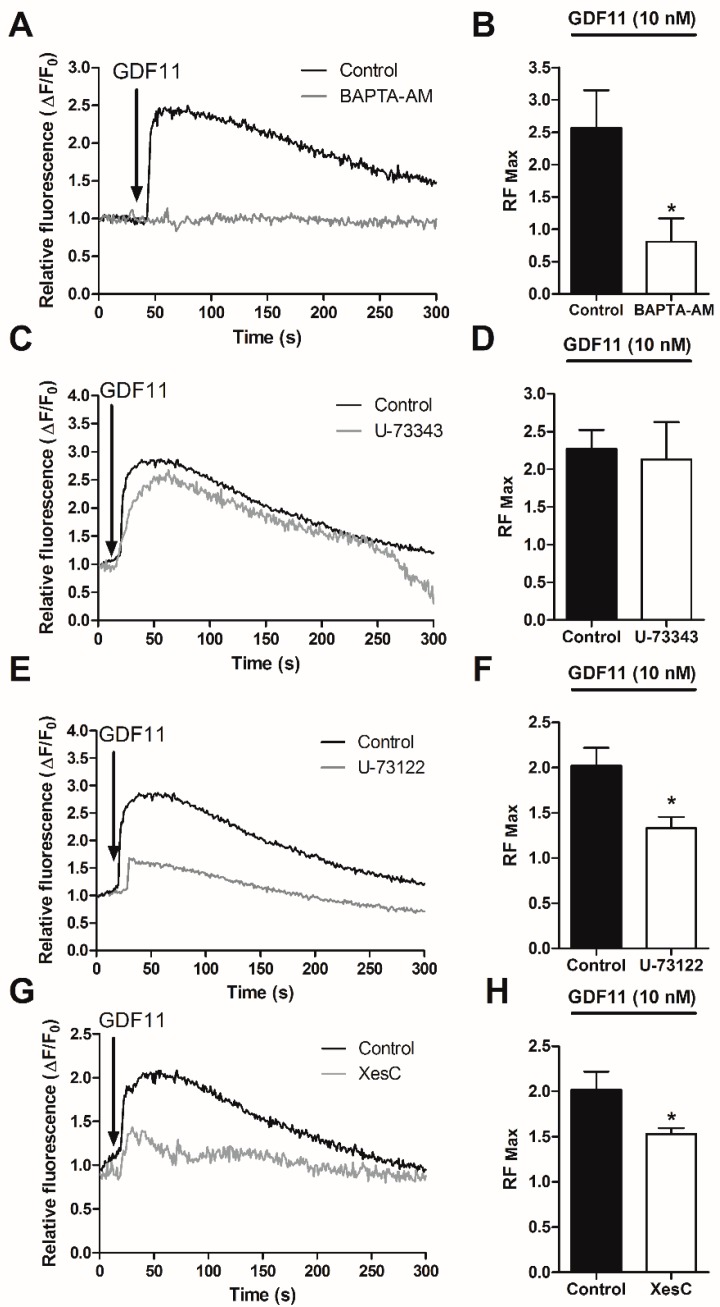
Effects of intracellular Ca^2+^ and the PLC/IP_3_R pathway on the intracellular Ca^2+^ increase induced by GDF11 in cultured cardiomyocytes. Cells were preloaded with Fluo3-AM and maintained in Ca^2+^-free medium at the moment of stimulation. Total ΔF/F_0_ calculated from fluorescence images of cardiomyocytes pre-incubated for 30 min with BAPTA-AM (100 µM, (**A**,**B**); U-73343 (50 µM, **C**,**D**); U-73122 (50 µM, **E**,**F**); or Xestospongin C (50 µM, **G**,**H**); and then stimulated with GDF11 (10 nM). Graphics correspond to the statistical analysis of RF max from experiments with the respective Ca^2+^ chelator or IP_3_ inhibitors and GDF11. Values are expressed as means ± SEM of triplicates from three independent experiments. Arrows indicate the time of addition of GDF11. * *p* < 0.05 versus the control.

To examine the intracellular Ca^2+^ sources involved in these signals, cardiomyocytes were pretreated for 30 min with 100 µM BAPTA-AM (an intracellular Ca^2+^ chelator) or with inhibitors of the IP_3_ pathway (50 μM U-73122, a phospholipase C (PLC) inhibitor; 50 μM U-73343, an inactive isomer of U-73122; and 50 μM Xestospongin C, an IP_3_ receptor inhibitor). After both Ca^2+^ quelation and IP_3_ pathway inhibition, the increase in Ca^2+^ in response to GDF11 (10 nM) was inhibited (Figure 2A–H), suggesting that the release of Ca^2+^ from intracellular stores evoked by GDF11 is mediated by the IP_3_ receptor (IP_3_R) pathway in cardiomyocytes.

### 2.2. GDF11 Activated Smad2/3 in Cardiomyocytes

**Figure 3:**
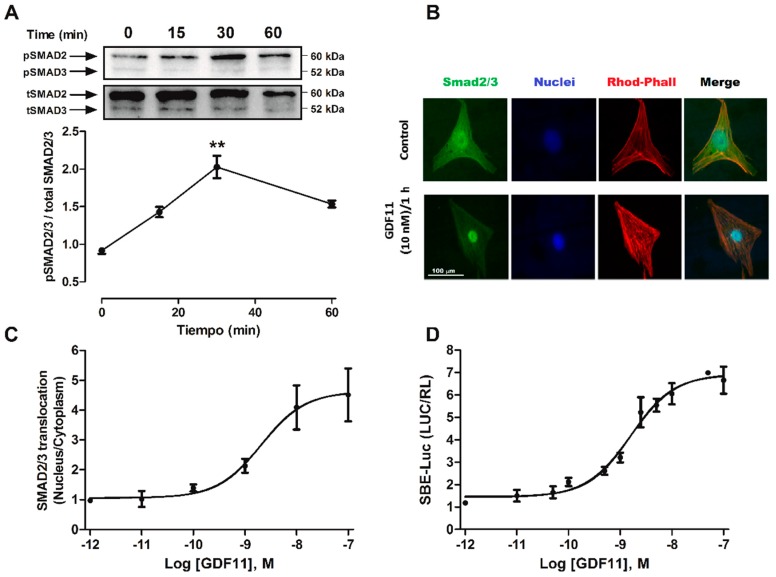
Effects of GDF11 on Smad2/3 phosphorylation, nuclear translocation, and SBE-Luc activity in cultured cardiomyocytes. (**A**) Cells were treated with GDF11 (10 nM) for different times (0–60 min) for Western blot analysis. The figure shows representative Western blots, and the graph shows relative phospho-Smad2/3 levels with respect to total Smad2/3 ratio; (**B**) Cells were stimulated with GDF11 (10 nM) for 60 min and then subjected to immunofluorescent staining with an anti-Smad2/3 antibody (green); nuclei were stained with Hoescht 33342 dye (blue); and sarcomeres were stained with phalloidin–rhodamine (red). The figure shows representative images for control and stimulated conditions; (**C**) Cells were treated with GDF11 for 1 h at different concentrations (1 pM to 100 nM) for immunocytochemistry experiments. Quantification of Smad2/3 staining is shown as the nuclear-to-cytoplasmic fluorescence ratio; (**D**) Cells were cotransfected with the plasmids SBE-Luc and Renilla-luciferase and stimulated with GDF11 for 24 h at different concentrations (1 pM to 100 nM). Smad2/3 activity was expressed as the SBE-Luc to Renilla luciferase ratio. Values are expressed as mean ± SEM of triplicates from three independent experiments. ** *p* < 0.01 versus control.

The TGF-β family, including myostatin/activin proteins, signals through the Smad2/3 pathway [26]. To evaluate whether GDF11 activated the Smad2/3 pathway in cardiomyocytes, we assessed the phosphorylation levels, nuclear translocation, and transcriptional activities of these proteins. First, we evaluated changes in the phosphorylation of Smad2 (Ser465/467) and Smad3 (Ser423/425) with specific antibodies. The dual phosphorylation of Smad2/3 represents a measurement of the enzyme complex activity [19]. In these experiments, cardiomyocytes were stimulated with GDF11 (10 nM) from 0 to 60 min. As shown in Figure 3A, GDF11 increased Smad2/3 phosphorylation, reaching a peak at 30 min of stimulation.

In a second approach to evaluate Smad2/3 activation, immunocytochemical studies were performed to determine cytoplasmic to nuclear cellular translocation of Smad2/3 proteins after stimulating cardiomyocytes with different concentrations of GDF11 (1 pM to 100 nM). The intracellular localization of the proteins was monitored by fluorescence microscopy, using specific antibodies targeting Smad2/3. As shown in Figure 3B, stimulation with GDF11 for 1 h increased the nuclear translocation of Smad2/3 in cardiomyocytes with a half-maximal response (EC50) of 2.08 nM (Figure 3C). Next, because nuclear translocation of Smad2/3 is necessary for its transcriptional activity, cardiomyocytes were transfected with the plasmids SBE-Luc and Renilla luciferase as a transfection control. The cardiomyocytes were stimulated with GDF11 at different concentrations (1 pM to 100 nM) for 24 h. GDF11 increased the transcriptional activity of SBE-Luc in a concentration-dependent manner with an EC50 of 1.06 nM (Figure 3D).

### 2.3. Effect of Ca^2+^-Dependent Pathways on Smad2/3 Activity Induced by GDF11 in Cardiomyocytes

**Figure 4:**
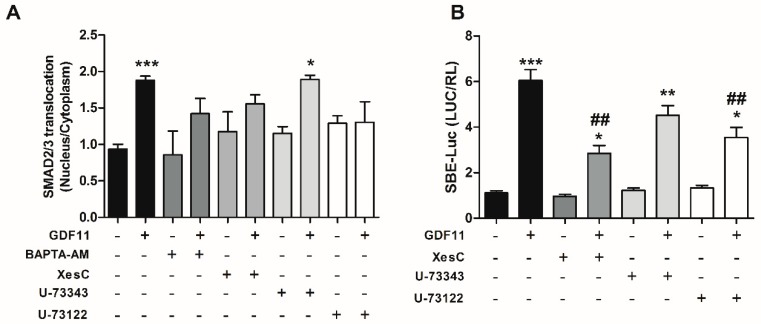
Effects of intracellular Ca^2+^ and the PLC/IP_3_R pathway on nuclear translocation and SBE-luc activity in cultured cardiomyocytes. (**A**) Cells were pretreated with BAPTA-AM (100 µM), U-73122 (50 µM), U-73343 (50 µM), or Xestospongin C (50 µM) and then stimulated with GDF11 (10 nM) for 1 h; (**B**) Cardiomyocytes were co-transfected with SBE-luc and Renilla luciferase plasmids; pretreated with U-73122 (50 µM), U-73343 (50 µM), or Xestospongin C (50 µM); and stimulated with GDF11 (10 nM) for 24 h. Values are expressed as mean ± SEM of triplicates from three independent experiments. * *p* < 0.05, ** *p* < 0.01, *** *p* < 0.001 versus the control; ## *p* < 0.01 versus GDF11.

To determine whether intracellular Ca^2+^ pathways were involved in Smad2/3 activation by GDF11, we examined whether nuclear translocation of Smad2/3 was dependent on Ca^2+^-dependent pathways. To this end, cardiomyocytes were treated with 100 μM BAPTA-AM, 50 μM U-73122, 50 μM U-73343, or 50 μM Xestospongin C and then stimulated with 10 nM GDF11 for 1 h. As shown in Figure 4A, we found that a Ca^2+^ chelator and inhibitors of the IP_3_ pathway inhibited Smad2/3 nuclear translocation, whereas the isomer inactive PLC inhibitor (U-73343) did not have significant effects, suggesting a role for intracellular Ca^2+^ and its dependent pathways on the translocation process.

Next, we investigated whether those Ca^2+^-dependent pathways were involved in SBE-Luc activity induced by GDF11. Accordingly, cardiomyocytes expressing SBE-Luc were pretreated with 50 μM U-73122, 50 μM U-73343, or 100 μM Xestospongin C and stimulated with 10 nM GDF11 for 24 h. GDF11 increased the activity of Smad2/3 in cardiomyocytes, and the use of PLC and IP_3_R inhibitors partially blocked the increase in Smad2/3 activity, whereas the inactive isomer of the inhibitor of PLC showed an increase similar to that induced only by GDF11 (Figure 4B). These results suggest that the PLC/IP_3_R pathway is involved in Smad2/3 activation induced by GDF11 in cardiomyocytes.

### 2.4. GDF11 Prevented Cardiomyocyte Hypertrophy Induced by Testosterone and Phenylephrine

**Figure 5:**
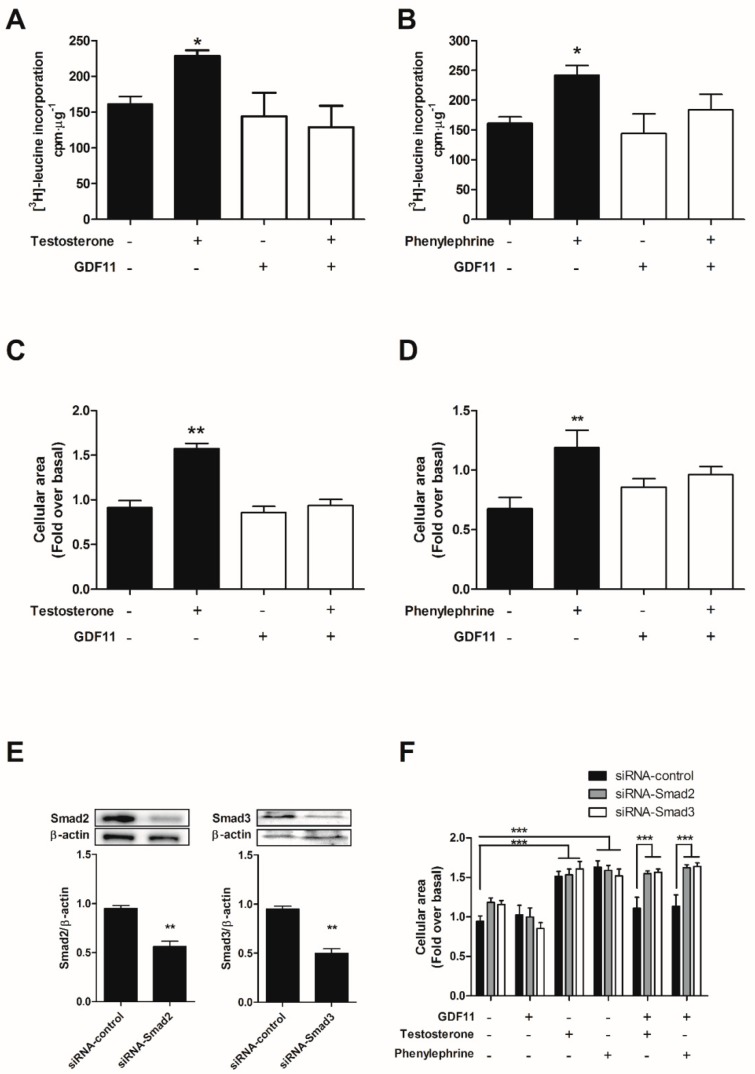
Effects of GDF11 and Smad2/3 on cardiomyocyte hypertrophy induced by testosterone and phenylephrine. Cells were pretreated with GDF11 (10 nM) for 24 h and stimulated with testosterone (100 nM) or phenylephrine (50 µM) for 48 h. (**A**,**B**) Leucine incorporation was assessed through [^3^H]-leucine (2.5 μCi/mL) pre-incubation; and (**C**,**D**) cellular area was assessed using the vital fluorescent dye CellTracker Green; (**E**) Cardiomyocytes were transfected with siRNA-control, siRNA-Smad2, or siRNA-Smad3, and protein levels were determined by Western blot; (**F**) Cellular area was measured in transfected cardiomyocytes stimulated with GDF11 (10 nM) for 24 h and stimulated with testosterone (100 nM) or phenylephrine (50 µM) for 48 h. For each condition, cellular area was quantified in 50–80 cells. Incorporation of [^3^H]-leucine was quantified using a liquid scintillation counter, and values are expressed as counts/min/μg protein. Values are expressed as mean ± SEM of triplicates from three independent experiments. * *p* < 0.05, ** *p* < 0.01, *** *p* < 0.001 versus the control.

To evaluate whether GDF11 exerted antihypertrophic effects, cardiomyocytes were exposed to various hypertrophic agents (100 nM testosterone or 50 μM phenylephrine) for 24 h. Prohypertrophic treatment increased cardiomyocyte size and [^3^H]-leucine incorporation, which was blunted by pretreatment with 10 nM GDF11 for 24 h before prohypertrophic treatment (Figure 5A–D). To investigate the involvement of Smad2/3 protein on the antihypertrophic effects produced by GDF11, cardiomyocytes were transfected with small interfering RNAs (siRNAs) for Smad2 or Smad3 (both 50 nM). Knockdown of both proteins significantly reduced protein content in cardiomyocytes (Figure 5E). Moreover, Smad2/3 knockdown abolished the antihypertrophic effects of GDF11 in both testosterone- and phenylephrine-induced cardiomyocyte hypertrophy (Figure 5F).

## 3. Discussion

In the present study, we determined that GDF11 induces intracellular Ca^2+^ increase through the IP_3_ pathway and activates Smad2/3 in cardiomyocytes. Furthermore, our findings suggest that pretreatment of cardiomyocytes with GDF11 prevented hypertrophy induced by testosterone and phenylephrine by modulating the Smad2/3 pathway.

In cardiomyocytes, effective intracellular Ca^2+^ signals and its fluctuations are related to excitation–contraction coupling and are intimately linked to diverse cellular processes, such as differentiation, cell metabolism, apoptosis, and hypertrophy [13,14]. In this study, we showed that GDF11 induced intracellular Ca^2+^ increases in a concentration-dependent manner in Ca^2+^-containing medium. Since GDF11 did not induce detectable contraction in cardiomyocytes we focused on the IP_3_ signaling pathway. To identify the Ca^2+^ release systems involved in these signals, we pharmacologically altered IP_3_-mediated processes pathways instead of ryanodine receptors related to the Ca^2+^-induced Ca^2+^ release mechanism. Ca^2+^ signals evoked by GDF11 were almost completely blocked by the use of inhibitors of the IP_3_ pathway, suggesting that IP_3_ was involved in GDF11-induced intracellular Ca^2+^ release in cardiomyocytes. In accordance with our results, other studies have reported that TGF-β members bind to their TFG-β receptor and promote Ca^2+^ release from intracellular stores [12,27]. In addition, this Ca^2+^ release is thought to be induced by the IP_3_ receptor, which could be involved in transcriptional regulation in cardiac cells [9,12].

Ca^2+^ activates several Ca^2+^-binding proteins and also regulates Ca^2+^-dependent pathways that mediate cardiomyocyte growth [9,10]. Our results indicate that Ca^2+^ participates in the activation of Smad2/3 induced by GDF11, which prevents hypertrophy induced by testosterone and phenylephrine, suggesting a common mechanism of action involved in the antihypertrophic effects of GDF11. Several reports have suggested that increased Ca^2+^ is a potential initiator of cardiac hypertrophy [14]. However, it remains unclear how Ca^2+^ is sensed by intracellular signaling pathways in cardiac cells given the dynamic changes in total Ca^2+^ levels, which are related to different processes, such as excitation–contraction coupling and excitation–transcription coupling [14]. Although Ca^2+^ has been largely described as a prohypertrophic second messenger, several reports have suggested that this effect depends on multiple factors, and that increase in intracellular Ca^2+^ is not sufficient to transduce hypertrophic signals. Indeed, different Ca^2+^ pools have distinct functions. Overexpression of the T-type Ca^2+^ channel subunit α1G in mice increases the Ca^2+^ current; however, these transgenic mice are resistant to cardiac hypertrophy. On the other hand, α1G^−/−^ mice enhance hypertrophy induced by isoproterenol and pressure overload, which activates guanosine 3′,5′-cyclic monophosphate (cGMP)-dependent protein kinase type I activity, an identified antihypertrophic protein [28] through nitric oxide synthase 3. Additionally, reports have indicated that overexpression of activators of caveolae-localized L-type Ca^2+^ channel microdomains increases the Ca^2+^ current but does not regulate hypertrophic signaling in the heart [29]. Hence, the sources of Ca^2+^ that activate either hypertrophic or antihypertrophic signaling have not been clearly defined yet, and several Ca^2+^ channels that increase Ca^2+^ influx could activate various processes such as contraction without affecting other responses, such as transcriptional effects. Thus, Ca^2+^ influx may not be prohypertrophic or antihypertrophic in cardiomyocytes. The nitric oxide pathway, through activation of cGMP-dependent protein kinase, prevents cardiac hypertrophy [30]. In several models, the IP_3_ pathway has been shown to be related to these signaling pathways [31], and protein kinase C, a protein that is classically activated by the IP_3_ pathway, is involved in mediating the effects of TGF-β on pancreatic cells [24].

The regulation of Smad2/3 by phosphorylation has been well documented [19]. Phosphorylation of Smad2/3 results from the activation of agonist-induced TGF-β receptor (activin receptors IIA and IIB (ActRIIA/B)) and activin-like receptor (ALK4/5/7) signaling cascades, permitting the translocation and binding of Smad3 to DNA. Different members of the TGF-β family, such as GDF15 or GDF8, activate both type II and I Ser/Thr kinase receptors. In addition, GDF11 and myostatin may also activate noncanonical (i.e., non-Smad) signaling, such as extracellular signal-regulated kinases 1/2, c-Jun N-terminal kinases, and p38 mitogen-activated protein kinase, thereby increasing the versatility of this mechanism of action [26]. Moreover, TGF-β members have been reported to exert their effects via intracellular Ca^2+^ release [32] or even through Ca^2+^-dependent pathways to mediate Smad2/3 activity [24,25]. Our findings showed that stimulation of cardiomyocytes with GDF11 caused an increase in Smad2/3 phosphorylation and its nuclear translocation. This enhancement of translocation was significant because most components of the Smad2/3 pathway reside in the nucleus, where Smad2/3, along with the Smad4 complex, acts as a transcription factor [33]. GDF15 is another member of the TGF-β superfamily; this protein is expressed in cardiac cells and activates Smad2/3. Transgenic mice that express GDF15 show normal phenotypes, but are resistant to cardiac hypertrophy induced by pressure overload [23]. GDF15 activates Smad2/3 in cultured cardiomyocytes, and the overexpression of Smad2 inhibits cardiomyocyte hypertrophy in a manner similar to that of GDF15, whereas the overexpression of Smad6/7 (antagonists of Smad2/3 action) inhibit the antihypertrophic effects of GDF15 following phenylephrine stimulation [23]. Additionally, ablation of Smad4 in mice generates cardiac hypertrophy and heart failure [33]. In this study, we found that GDF11 increased Smad2/3 activity and prevented cardiomyocyte hypertrophy with the involvement of Smad2/3 proteins through intracellular Ca^2+^-dependent pathways. Salvarani et al. (2017) reported that TGF-β1 induces depolarization of cardiomyocytes coupled to myofibroblasts, but not in cardiomyocyte–cardiomyocyte gap junctional coupling. The actions of GDF11 on cardiac resting membrane potential can be of upmost importance, especially in the context of improving the function of a failing heart during aging, considering that the amount of myofibroblasts increases [34].

The data presented here are consistent with other reports describing the antihypertrophic effects of GDF11 both in vivo and in vitro [6,20]. GDF11 levels are increased in hearts exposed to ischemia/reperfusion, and exogenous administration of GDF11 decreases the infarcted area [3]. Moreover, circulating levels of GDF11 decrease with age [6,20], and supplementation with GDF11 in aged mice reverses cardiac hypertrophy, enhances the dysfunction and genomic stability of satellite cells and the functional and structural characteristics of skeletal muscle [35], as well as promoting neurogenesis and olfactory discrimination [21]. In contrast, other reports have contradicted these findings by showing that GDF11 circulating levels were not altered with age and that GDF11 levels increased with age [7]. Two reports showed that GDF11 did not reverse age-related pathological hypertrophy [7] and decreased basal cardiac mass in mice [4]. Another report suggested that higher GDF11/myostatin circulating levels were associated with a lower risk of cardiovascular events and death [5], and a separate study demonstrated that GDF11 did not decline with age and was associated with a higher risk of cardiovascular diseases [36].

Moreover, other reports have indicated that GDF11 induces cachexia [37], produces deleterious effects on muscular regeneration, and decreases satellite cell expansion in old mice [8]. More recent studies have characterized the effects of GDF11 in multiple in vivo and in vitro models. For example, GDF11 administration decreases apoptosis, increases the mass and function of β cells in pancreatic islets in a model of obesity [38], and enhances renal function induced by ischemia/reperfusion injury through the dedifferentiation and proliferation of epithelial cells in proximal tubules, suggesting improvements in renal function in elderly mice [39]. However, GDF11 has not been shown to be related to the enhancement of insulin resistance induced by palmitate in a skeletal muscle cell line [40] or to lifespan in a premature elderly animal model [41]. Therefore, given the aforementioned discrepancies in the results of different groups, further studies are needed to elucidate the signaling mechanisms of GDF11 in cardiomyocytes.

## 4. Materials and Methods

### 4.1. Isolation and Culture of Cardiomyocytes from Neonatal Rats

All procedures with animals were approved by the Bioethics Committee of the Faculty of Medicine at University of Chile (Protocol CBA 0451 FMUCH, March 2015). Cells were isolated and cultured using a protocol similar to that described in a previous report [42]. Ventricular cardiomyocytes were obtained from hearts of 1–3-day-old neonatal Sprague Dawley rats. Briefly, fresh cardiac tissue was exposed to enzymatic digestion, and isolated cells were cultured in 35-mm petri dishes precoated with gelatin. Cells were plated at high and low densities for biochemical analyses or Ca^2+^ imaging experiments. Immunodetection studies used 35-mm plates seeded at a density of 1 × 10^6^ cells/culture plate. For luciferase activity and microscopy, the cells were used at densities of 1.5 × 10^6^ and 2.5 × 10^5^ cells/wells, respectively.

### 4.2. Measurement of Intracellular Ca^2+^

Ca^2+^ imaging and fluorescence measurements using Fluo-3-AM fluorescent dye were carried out as previously described [43]. The images were recorded at intervals of 5 s using standard 2.6 mM Ca^2+^ medium (145 mM NaCl, 2.6 mM CaCl_2_, 5 mM KCl, 1 mM MgCl_2_, 10 mM HEPES-Na, 5.6 mM glucose, pH 7.4). CaCl_2_ was substituted with 1 mM EGTA to prepare Ca^2+^-free extracellular medium. Fluorescent images were acquired using a Zeiss–Colibri epifluorescence microscope (Zeiss, Oberkochen, Germany) and analyzed using ImageJ software from the National Institutes of Health (NIH, Bethesda, MD, USA). Ca^2+^ levels were determined as the ratio of stimulated intensity relative to basal fluorescence. Intracellular Ca^2+^ levels were expressed as relative total fluorescence (ΔF/F_0_: ratio of fluorescence difference, stimulated-basal (Fi–F_0_), to basal value (F_0_)) as a function of time and maximal relative fluorescence (RFmax) was calculated using Origin pro 8 software (OriginLab Corporation, Northampton, MA, USA).

### 4.3. Transfections and Reporter Assay

To determine the transcriptional activity of the Smad2/3 pathway, cardiomyocytes were transfected with the Smad2/3-responsive luciferase reporter plasmid SBE-Luc (provided by Bert Vogelstein; Addgene #16527) which contain 4 Smad-binding boxes cloned upstream of the firefly luciferase reporter gene [44,45], and the Renilla luciferase plasmid (internal control for transfection efficiency) and the results were expressed as the ratio of SBE-Luc/RL. siRNAs were used to decrease Smad2/3 protein levels. Transfections were carried out using Lipofectamine RNAiMAX (Invitrogen, Carlsbad, CA, USA) and Opti-MEM Reduced Serum Medium, GlutaMAX Supplement (Thermo-Fisher Scientific, Rockford, IL, USA) according to the manufacturer’s specifications. For each condition, final concentrations of 1 μg/mL SBE4-Luc plasmid, 50 nM siRNA, and 0.5 μg/mL Renilla-luciferase were used. The effectiveness of the siRNA for Smad2/3 was verified by immunodetection using specific antibodies for total protein content. After the experiments, cells were lysed, and SBE4-Luc and Renilla luciferase activities were measured using a Dual-Luciferase Kit Assay Reporter System (Promega, Madison, WI, USA) and a luminometer (Berthold luminometer F12; Pforzheim, Germany).

### 4.4. Western Blotting

For immunoblot analyses, cell lysates were homogenized and centrifuged at 15,000 rpm for 20 min. The supernatant containing the proteins was extracted and quantified using a Rapid Gold BCA Protein Assay Kit (Thermo-Fisher Scientific, Rockford, IL, USA). Equivalent amounts of protein (20 μg from cardiomyocyte extracts) were mixed with sample buffer, boiled, and separated by electrophoresis on 7% sodium dodecyl sulfate polyacrylamide gels. Proteins were transferred to nitrocellulose Hybond membranes (Amersham Biosciences Corporation, Piscataway, NJ, USA) with Pierce G2 Fast Blotter (Thermo-Fisher Scientific, Rockford, IL, USA). The membranes were maintained in blocking solution (TBS containing 0.2% Tween and 2.5% bovine serum albumin (BSA)) for 1 h and then incubated overnight at 4 °C with the following primary antibodies in blocking solution: anti-phospho-Smad2 (Ser465/467), anti-phospho-Smad3 (Ser423/425), anti-total Smad2 and Smad3 (1:1000; Cell Signaling Technology, Danvers, MA, USA), and anti-β-actin (1:5000; Sigma, St. Louis, MO, USA). The membranes were then washed and incubated with specific secondary antibodies (anti-mouse or anti-rabbit, 1:5000) conjugated to peroxidase for 1–2 h at room temperature. Immunoreactive bands in the blots were detected using SuperSignal West Pico Chemiliminescent Substrate (Thermo-Fisher Scientific) and Westar Supernova (Cyabagen, Bologna, Italy) on a ChemiDoc Imagen System (Bio-Rad, Hercules, CA, USA). For the densitometric measurement of band intensity, the images were scanned and analyzed using ImageJ software (NIH). After initial probing, membranes were stripped with Restore PLUS Western Blot Stripping Buffer (Thermo-Fisher Scientific) for 20 min at room temperature, and the incubation with primary antibody was repeated for protein loading normalization using anti-β-actin antibodies.

### 4.5. Immunocytochemistry

Cardiomyocytes were seeded onto coverslips (12 mm in diameter) at 25%–50% confluence. Cells were rinsed in phosphate-buffered saline (PBS; 10 mM Tris (pH 7.5), 100 mM NaCl, 5 mM KCl) and then fixed with 4% paraformaldehyde for 10 min at 4 °C. Following two washes with PBS, cells were permeabilized with 0.2% Triton X-100 in PBS for 10 min at room temperature. Cells were then rinsed and incubated with blocking solution (5% BSA in PBS) for 1 h. The cells were then incubated overnight at 4 °C with primary antibody diluted in blocking solution. Anti-Smad2/3 antibodies (Cell Signaling Technology) were used at a 1:100 dilution. Cells were rinsed three times with PBS prior to the incubation with secondary antibody conjugated to Alexa Fluor 488 diluted in blocking solution for 1 h. For nuclear and sarcomere staining, the cells were incubated with Hoescht 33342 dye (1:400; Thermo-Fisher Scientific) and phalloidin-rhodamine (1:400, Sigma) for 5 min at room temperature. Finally, the coverslips with the cells were mounted in DAKO medium (Sigma) for 5 min. The cells were visualized using a Zeiss–Colibri epifluorescence microscope (Zeiss) with a 40× objective. Quantification of the fluorescence was performed using ImageJ software (NIH). To quantify fluorescence, the summed pixel intensity was calculated for each section delimited by a region of interest (ROI) within either nucleus or cytoplasm of cardiomyocytes.

### 4.6. Incorporation of Amino Acids

Cardiomyocytes were seeded onto 24-well plates at a density of 4 × 10^5^ cells/mL and were pretreated with or without 10 nM GDF11 for 24 h, followed by hypertrophic treatment with testosterone (100 nM) or phenylephrine (50 µM) for 48 h. To quantify the incorporation of amino acids, the cells were previously incubated with [^3^H]-leucine (2.5 μCi/mL; New England Nuclear-PerkinElmer, Wellesley, MA, USA). The cells were lysed, treated with 10% trichloroacetic acid, and centrifuged at 15,000× *g* rpm. The resulting pellet was dissolved in NaOH (0.2 M). Radioactivity of the cells was evaluated using a LS&500 liquid scintillation counter (Beckman Coulter, Brea, CA, USA) to obtain the radioactive counts per minute (CPM). The CPM for each experimental condition in triplicate was obtained using a scintillation counter and normalized to the concentration of proteins in each sample.

### 4.7. Cell Size

Cardiomyocytes were pretreated with GDF11 (10 nM) for 24 h and stimulated with testosterone (100 nM) or phenylephrine (50 µM) for 48 h. To perform cell size measurements, the cells were washed with PBS buffer and incubated for 30 min with Cell Tracker Green (10 μM; Molecular Probes, Invitrogen). Next, the cells were washed with PBS and fixed with 4% paraformaldehyde for 15 min at room temperature. A Zeiss–Colibri epifluorescence microscope (Zeiss) and ImageJ software (NIH) were used to determine cardiomyocyte surface area. Five observation fields were selected randomly on each cover slip, and 5–8 cells within each observation field were selected for determination of the mean cardiomyocyte surface area according to the image analysis system.

### 4.8. Statistical Analysis

All data are presented as mean ± standard error of the mean (SEM). Comparisons of two groups were analyzed by Student’s *t* tests, and comparisons between several means were analyzed by one-way analysis of variance, followed by Tukey’s post-hoc test using GraphPad Prism 6 (GraphPad Software Inc., San Diego, CA, USA). Differences with a *P* value of less than 0.05 were considered statistically significant.

## 5. Conclusions

In contrast to the well-established role of Ca^2+^ during cardiomyocyte hypertrophy, little is known about the involvement of Ca^2+^ in antihypertrophic pathways. In the present study, we determined that GDF11 induces intracellular Ca^2+^ increase mediated through the IP_3_ pathway and activates Smad2/3 to modulate cardiomyocyte hypertrophy. It will be interesting to study the mechanisms through which Ca^2+^ is involved in the regulation of Smad2/3 activity and to determine the sources of Ca^2+^ mediated by GDF11 in cardiomyocytes. Additionally, further studies are needed to determine whether GDF11 prevents hypertrophy through a Smad2/3-dependent pathway or whether this protein can activate other antihypertrophic pathways (such as the glycogen synthase kinase-3β pathway) or inhibit prohypertrophic pathways (such as the nuclear factor of activated T cells pathway). Improving our understanding of these pathways will be useful to elucidate the mechanisms of GDF11 in cardiomyocytes, increase our knowledge of the signaling pathway of TGF-β family members, and characterize the regulation of anti- and prohypertrophic pathways in the heart.
